# Myocardial native T2 measurement to differentiate light-chain and transthyretin cardiac amyloidosis and assess prognosis

**DOI:** 10.1186/s12968-018-0478-3

**Published:** 2018-08-16

**Authors:** Fourat Ridouani, Thibaud Damy, Vania Tacher, Haytham Derbel, François Legou, Islem Sifaoui, Etienne Audureau, Diane Bodez, Alain Rahmouni, Jean-François Deux

**Affiliations:** 1 Radiology Department, Henri Mondor Hospital, University Paris Est Créteil, Assistance Publique-Hôpitaux de Paris, 51 av Mal de Lattre de Tassigny, 94000 Créteil, France; 2 Cardiology Department, Henri Mondor Hospital, University Paris Est Créteil, Assistance Publique-Hôpitaux de Paris, Créteil, France; 3 National Referal Centre for Cardiac Amyloidoses, Henri Mondor Hospital, Créteil, France; 4 Public Health Department, Henri Mondor Hospital, CEpiA EA7376, University Paris Est Créteil, Assistance Publique-Hôpitaux de Paris, Créteil, France

**Keywords:** Amyloidosis, CMR, T2 mapping

## Abstract

**Background:**

To assess the diagnostic and prognosis value of myocardial native T2 measurement in the distinction between Light-chain (AL) and Transthyretin (ATTR) cardiac amyloidosis (CA).

**Methods:**

Forty-four patients with CA (24 AL; 20 ATTR) and 40 healthy subjects underwent 1.5 T cardiovascular magnetic resonance (CMR). They all underwent T1 and T2 mapping (modified Look-Locker inversion recovery), cine and late gadolinium enhancement (LGE) imaging. The Query Amyloid Late Enhancement (QALE) score, myocardial native T2, T1 and extra cellular volume fraction (ECV) were calculated for all patients.

**Results:**

Of the 44 patients, 36 (82%) exhibited enhancement on LGE images. Mean QALE score of AL (7.9 ± 6) and ATTR (10.5 ± 5) patients were similar (*p* = 0.6). Myocardial native T2 was significantly (*p* < 0.0001) higher in AL (63.2 ± 4.7 ms) than in ATTR (56.2 ± 3.1 ms) patients, and both higher (*p* < 0.001) than healthy subjects (51.1 ± 3.1 ms). Myocardial native T2 was highly correlated with myocardial native T1 (Spearman’s rho = 0.79; p < 0.001) and exhibited higher diagnostic performance than T1 to separate AL and ATTR patients: the area under curve (AUC) of T2 was 0.94 (95% CI: 0.86–1, p < 0.001) and the AUC of T1 was 0.77 (95% CI: 0.62–0.91, *p* = 0.03). Myocardial native T2 did not impact overall survival in patients (HR 1.03 (0.94–1.12); *p* = 0.53) in contrast to ECV that was the best predictor of outcome (HR 1.66 per 0.1 increase in ECV (1.24–2.22); *p* = 0.0006).

**Conclusions:**

Myocardial native T2 significantly is increased in CA, especially in AL patients in comparison to ATTR patients. Myocardial native T2 does not impact survival in CA patients in contrast to ECV that was the best predictor of outcome.

**Trial registration:**

Trial Registration and unique number: CNIL cardio 1778041. Date of registration: 20 December 2012.

## Background

Cardiac amyloidosis (CA) is characterized by interstitial amyloid infiltration which leads to progressive thickening of cardiac walls, diastolic dysfunction, and restrictive cardiomyopathy [1–3]. Cardiac involvement in systemic amyloidosis leads to a progressive, fatal, cardiac failure and is an important factor in treatment options and prognosis [4]. Diagnosis of CA may be challenging because clinical symptoms, cardiac biomarkers, and transthoracic echocardiographic abnormalities are nonspecific, especially when other causes of hypertrophy exist [5–7]. Moreover, they often appear at a late stage of the disease. Cardiovascular magnetic resonance (CMR) imaging has been reported a promising tool to detect cardiac involvement [8]. Late gadolinium enhanced (LGE) sequence shows either diffuse or circumferential enhancement after gadolinium injection that is highly specific to CA [9–11]. The measurement of myocardial native T1 and extracellular volume fraction (ECV) using parametric sequences can also provide useful additive insights for the diagnosis [12] that could be very useful when gadolinium infusion is contra-indicated. Therefore, CA is associated with a significant rise in myocardial native T1 and ECV [13, 14]. These parameters could be considered as early diagnostic markers [15] useful to predict mortality [16]. In contrast to myocardial native T1, myocardial T2 relaxation time variations have been less often evaluated in CA, and controversial findings were reported [17–19].

Although CMR consistently detects CA, diagnosis of the types of amyloidosis, in particular the distinction between light chain (AL) and transthyretin (ATTR) amyloidosis, may be challenging [20]. In most cases, ATTR had a more increased left ventricular (LV) mass, a thicker interventricular septum (IVS), larger atrial areas, smaller cavity volumes and a lower LV ejection fraction (LVEF) than AL amyloidosis [8]. Myocardial enhancement on LGE sequence is reported to be more intense in ATTR than in AL amyloidosis, with predominant transmural enhancement and frequent right ventricular (RV) involvement [21]. More recently, using parametric imaging, Fontana et al. reported that ATTR amyloid deposits were larger than AL amyloid deposits and were associated with a ~ 20% increase in cell volume [22], suggesting a concomitant myocyte hypertrophy. AL amyloidosis was associated with a greater elevation of myocardial native T1 and a smaller ECV suggests myocardial edema. However, T2 mapping sequences were not performed to confirm this hypothesis. In this study, we hypothesized that in CA the myocardial edema represented a significant component of cardiac involvement with a different intensity between AL and ATTR patients. We used native T1 and T2 mapping parametric sequences to detect edema in those patients, and to evaluate relationships between parametric data and biological parameters, as well as patients’ survival.

## Materials and methods

### Study population

The study was approved by our local Research Ethics Committee and included written informed consent from each subject. Forty-four consecutive patients with CA (AL, *n* = 24, ATTR, *n* = 20, 9 mutant and 11 wild type) who underwent CMR between January 2013 and December 2014 at our center were retrospectively identified. Diagnosis and the type of systemic amyloidosis was biopsy-proven for all patients (labial or endomyocardial biopsy (EMB)). For patients with AL amyloidosis, cardiac involvement was histologically confirmed by EMB performed 1.2 (0–6) months after CMR. For the patients with ATTR amyloidosis, diagnosis of CA was based on significant heart retention of 99mTc-HMDP bone tracer on scintigraphy (*n* = 20; 100%) as recently proposed in the literature [23]. TTR genetic testing was obtained for all ATTR amyloidosis individuals: 5 (56%) patients carried Val30Met TTR mutation and the remaining 4 (44%) mutant ATTR patients carried other mutations; wild-type ATTR amyloidosis patients had no TTR mutation.

For all patients, NT-proBNP, troponin T and creatinine (μmol/L) levels, and echocardiography parameters (IVS thickness, LVEF, transmitral E/E’ ratio and global longitudinal strain) were recorded at the time of CMR as previously described [24]. A control group of 40 healthy subjects (20 males; 40 ± 12 years) was also included in the study and explored with CMR. Echocardiography parameters (IVS thickness, LVEF and global longitudinal strain) and levels of creatinine were recorded for all healthy subjects as well.

### CMR imaging

#### Acquisition protocol

All patients and healthy subjects were explored using the same CMR protocol except for post contrast imaging sequences that were not performed for the latter. CMR imaging was performed with a 1.5 T CMR system (Magnetom Avanto, Siemens Healthineers, Erlangen, Germany) equipped with a high-performance gradient sub-system (maximum amplitude, 40 mT/m; minimum rise, 200 μs), and an 8-channel phased-array cardiac coil. Unenhanced cine balanced steady state free precession (bSSFP) sequences, acquired in the LV short-axis section and encompassing the entire LV were performed on all patients. The following parameters were used: TR/TE, 2.8/1.4 (apparent TR, 31.4 ms; 11 segments); flip angle, 82°; matrix size, 192 × 192; FOV, 300 × 270 mm; slice thickness, 8 mm. Retrospective electrocardiogram (ECG) gating was used with 25 phases per section.

T1 maps were acquired before injection (myocardial native T1) and 15 min after gadolinium administration (0.2 mmol/Kg of gadolinium (Dotarem; Guerbet; Aulnay-sous-Bois; France)) in all the patients in a middle short-axis and in the four-chamber planes using the modified Look-Locker inversion recovery (MOLLI) sequence [25]. The following parameters were used: 3 inversion sets of 3/3/5 images, TE/TR = 1.06/2.5 ms, nominal flip angle = 35°, TI_1_ = 100 ms, DTI = 80 ms, matrix = 192 × 154, FOV = 340 × 274 mm^2^, BW = 930 Hz/pixels, slice thickness = 8 mm, generalized autocalibrating partially parallel acquisition (GRAPPA) 2 with 36 separated reference lines, 75% of partial Fourier, 3 R-R cycles recovery period and an acquisition time = 17 R-R cycles. T2 maps were generated using a non-product bSSFP sequence with an adiabatic T2 preparation, and acquired at the same location as T1 maps. The following parameters were used: TE/TR = 1.12/2.6 ms, 3 T2-preparation times = 0/25/55 ms, matrix = 192 × 154, FOV = 340 × 154, BW = 930 Hz/pixels, slice thickness = 6 mm, GRAPPA 2 with 36 separated reference lines, 75% of partial Fourier, acquisition time = 12 R-R cycles. All images were acquired within a single breath hold. A fast variational non-rigid registration algorithm was used to correct for residual cardiac and respiratory motion between images, aligning all T1- and T2-prepared frames to the center frame. Finally, T1 and T2 maps were generated from these motion-corrected images by fitting a mono-exponential decay curve at each pixel. A short Tau inversion recovery (STIR) T2-weighted image was acquired in the middle short axis section at the same level as T1 and T2 maps using the following parameters: TE/TR = 49/1500 to 2500 ms (depending on the heart rate), matrix = 192 × 154; field-of-view (FOV) = 340 × 154, BW = 255 Hz/pixel, slice thickness = 8 mm, turbo factor 15 and TI 150 ms. A surface coil intensity correction algorithm was used to compensate the myocardial intensity inhomogeneity.

LGE images covering the LV in short-axis and long-axis views were obtained 10 min after injection of 0.2 mmol/Kg of gadolinium (Dotarem; Guerbet; Aulnay-sous-Bois; France) in all the patients. A segmented 3D IR gradient-echo T1-weighted sequence was used with the following parameters: repetition time of 3.9 ms, echo time of 1.4 ms, flip angle of 10°, matrix size of 192 × 192, FOV 300 × 270 mm, 12 sections, and 6 mm slice thickness. Image acquisition lasted between [12 to 20 s] depending on the heart rate. A dedicated inversion recovery time (TI) scouting sequence was used before acquisition of LGE images to adjust the optimal TI. Phase sensitive inversion recovery (PSIR) images were systematically acquired after acquisition of LGE images because suboptimal nulling of the myocardial signal may be encountered in CA [26]. Sequence parameters of the PSIR sequence were as follows: repetition time of 835 ms, echo time of 3.3 ms, flip angle of 10°, matrix size of 256 × 156, FOV 300 × 270 mm, and 8 mm slice thickness. Image acquisition lasted between 8 to 12 s, depending on the heart rate. Five PSIR images were acquired in the short-axis plane encompassing the LV. One slice was also acquired in the 4-chamber and in the 2-chamber view.

### Image analysis

#### Qualitative analysis

Two reviewers, with 15 (JFD) and 3 (FR) years of experience in cardiovascular imaging, analyzed anonymously CMR images of all patients in consensus on a dedicated acquisition platform (Leonardo; Siemens Healthineers). The readers were blinded to the clinical data. LGE were scored on a five-point scale: 0: no abnormal enhancement detected within cardiac chambers, 1: doubtful enhancement within LV, 2: subendocardial enhancement of LV, 3: diffuse enhancement of LV, 4: patchy enhancement. Abnormal enhancement of RV and atria was also noted. Myocardial enhancement was also evaluated using the Query Amyloid Late Enhancement (QALE) score as previously reported [21]. The QALE score was performed on LGE images at the base, mid ventricle and apex in the LV and RV. Each LV level is scored according to the degree of LGE, with the highest score for circumferential and transmural LV LGE. The QALE score range for the whole heart in each patient is from 0 (no detectable LGE in the LV or RV) to 18 (global transmural LV LGE at all 3 levels plus RV involvement).

#### Quantitative analysis

The IVS was measured for all subjects on the end-diastolic short-axis cine image acquired at the middle part of the LV. LV indexed end-diastolic volume, indexed end-systolic volume, LVEF and indexed cardiac mass were calculated on cine short-axis images using a dedicated software (cmr42; Circle Cardiovascular Imaging Inc.; Calgary; Alberta; Canada). For myocardial native T1 and T2 measurements, the mid-ventricular short-axis and the 4 chamber images were manually contoured to outline the endocardium and epicardium, and the average T1 and T2 values were calculated for all patients using a dedicated software (cmr42). Same measurements were performed on post contrast T1 mapping images. In addition, T1 values of blood pool before and after contrast administration were calculated from a region-of-interest (ROI) placed in the blood pool cavity on the middle short-axis section. ECV was calculated using the following formula: ECV = λ (1- hematocrit), where λ = [(1/T1 myocardium _post-Gd_) – (1/T1 myocardium _pre-Gd_)] / [(1/T1 blood pool _post-Gd_) – (1/T1 blood pool _pre-Gd_)]. Intra cellular volume (ICV) was calculated as follows: ICV = 1 – ECV. Lastly, Total amyloid volume and Total cell volume were calculated using the following formulas:

Total amyloid volume (mL/m^2^) = ECV x LV volume.

Total cell volume (mL/m^2^) = ICV x LV volume.

T2 ratio was calculated by dividing signal intensity of LV myocardium (obtained from manual contouring of endocardial and epicardial LV boundaries of short-axis T2 STIR image) and signal intensity of chest wall muscle.

### Follow up and survival

Follow-up began at completion of the CMR. Dates and status of death were obtained from the medical records; as needed, details were obtained by contacting the referring physician.

### Statistical analysis

Quantitative data are expressed as means ± SD or medians and interquartile (IQR) depending on the normality of the data. Categorical variables were described as number (%) and compared using the χ^2^ or the Fisher’s exact test, as appropriate. Differences between continuous data were tested using unpaired t-test or Mann–Whitney rank sum test for two groups comparisons, and analysis of variance (ANOVA) or Kruskal-Wallis for three groups comparisons followed by post-hoc pairwise comparisons with Bonferroni correction in case of global significance, as appropriate. Spearman’s rank correlation coefficients were calculated to assess correlation between continuous variables. Receiver operating characteristic (ROC) analysis, with corresponding measures of statistical uncertainty (i.e., 95% confidence intervals), was applied to myocardial native T1 and T2 and QALE score to identify optimal cut-off values for cardiac involvement based on the highest Youden index. Sensitivity and specificity were calculated using the thresholds previously defined. Overall survival was measured from the date of CMR to the date of death or last follow-up. Unadjusted survival curves were plotted by the Kaplan-Meier method, using log-rank tests to assess significance for group comparison. Unadjusted Cox proportional hazards regression models were performed to compute hazard ratios (HR) along with their 95% confidence intervals. Based on Schoenfeld residuals, all predictors were tested for the proportional-hazards assumption which was not found to be violated. For continuous variables, optimal thresholds for overall survival were determined using recursive partitioning analysis (RPA), based on the martingale residuals from a Cox model to determine the optimal value among all possible cut-points. Data were considered significant if *p* < 0.05. Analyses were performed using SPSS 25.0 (International Business Machines, Armonk, New York, USA) and Stata v14.1 (StataCorp, College Station, Texas, USA), using an implementation of RPA for Stata by Wim van Putten [27].

## Results

### Population characteristics

Patients with ATTR amyloidosis were older compared with AL amyloidosis patients (73 vs. 65 years; *p* = 0.04) and predominantly male (85% vs. 58%; p = 0.04). No significant differences were detected between AL and ATTR patients regarding biological and echocardiographic parameters. Healthy subjects were significantly younger than AL (*p* < 0.001) and ATTR (*p* < 0.001) patients and did not exhibit cardiac hypertrophy. They had higher LVEF than AL (*p* < 0.001) and ATTR (*p* < 0.001) patients. All data are reported in the Table 1.

**Table 1:** Population characteristics

Characteristics	AL (n = 24)	ATTR (*n* = 20)	Healthy subjects (*n* = 40)	p*
Clinical				
Age (years)	65 **±** 12	73 **±** 15	40 **±** 12	0.04^$^
Male (%)	14 (58)	17 (85)	20 (50)	0.04
BMI (kg/m^2^)	24.7 **±** 3	24.5 ± 4	22.1 **±** 3	0.2
Diabetes	2	5	–	0.1
NYHA(I/II/III/IV)	3/9/9/3	2/8/9/1	–	0.6
Hypertension	9	13	–	0.1
Hyperlipidemia	9	9	–	0.9
NT-pro BNP (pg/mL)	6317 (340–18,908)	2384 (542–9129)	–	0.3
Troponin T (pg/mL)	52 (14–112)	38 (8–54)	–	0.5
Creatinine (μmol/L)	110 (78–275)	101 (89–167)	63 (55–77)	0.6^$^
Echocardiography				
Septal thickness (mm)	17 ± 5	18 ± 9	8 ± 4	0.4^$^
LVEF (%)	57 ± 15	50 ± 16	66 ± 15	0.2^$^
Transmitral E/A	3.6 ± 8	2.6 ± 8	–	0.2
E/E’	16.3 ± 8	15.5 ± 8	–	0.9
GLS (−%)	12 (6–17)	9 (8–15)	18 (15–22)	0.8^$^

*AL* light chain amyloidosis; *ATTR* Transthyretin amyloidosis; *BMI* Body Mass Index; *GLS* Global longitudinal strain; *LVEF* left ventricular ejection fraction; *NT-proBNP* N-terminal pro-B-type natriuretic peptide; *NYHA* New York Heart Association

*: AL vs. ATTR patients; $: *p* < 0.001: Healthy subjects vs. CA patients

### CMR images analysis

#### Qualitative analysis

Thirty-six (82%) patients exhibited enhancement of the LV on LGE images, described as subendocardial (grade 2) in 10 patients (23%; 5 AL and 5 ATTR), diffuse (grade 3) in 22 patients (50%; 10 AL and 12 ATTR) and patchy (grade 4) in 4 patients (9%; 3 AL and 1 ATTR). Six patients (14%; 4 AL and 2 ATTR) were considered as doubtful (grade 1) and 2 patients (4.5%; 2 AL) were considered negative (grade 0). RV involvement was suspected in 21 patients (48%; 11 AL and 10 ATTR) and atrial involvement was suspected in 21 patients (48%; 14 AL and 7 ATTR). Mean QALE score of AL (7.9 ± 6) and ATTR (10.5 ± 5) patients did not exhibit significant difference (*p* = 0.6).

#### Quantitative analysis

LV mass was significantly (*p* < 0.05) higher in ATTR than in AL patients. Healthy subjects had significantly (*p* < 0.001) higher LVEF than AL and ATTR patients, and did not exhibit cardiac hypertrophy. All data are reported in the Table 2.

**Table 2:** Comparison of cardiac MRI parameters between groups

MRI parameters	AL (*n* = 24)	ATTR (n = 20)	Healthy subjects (n = 40)	p*
LV EDV (mL/m^2^)	74 ± 23	89 ± 27	77 ± 16	0.06^£^
LV ESV (mL/m^2^)	35 ± 21	47 ± 28	27 ± 9	0.1^$^
IVS (mm)	17 ± 3	18 ± 3	9 ± 2	0.3
LV mass (g/m^2^)	97 ± 28	115 ± 31	63 ± 11	0.04^$^
LVEF (%)	56 ± 18	50 ± 19	65 ± 8	0.3^$^
Native T1 (ms)	1104 ± 54	1066 ± 42	975 ± 26	0.01^$^
Native T2 (ms)	63.2 ± 4.7	56.2 ± 3.1	51.1 ± 3.1	0.0001^$^
T2 ratio	1.31 ± 0.4	1.41 ± 0.2	1.44 ± 0.3	0.2
Post contrast T1 (ms)	378 ± 73	363 ± 69	NA	0.9
ECV	0.53 ± 0.17	0.46 ± 0.11	NA	0.2
ICV	0.47 ± 0.17	0.54 ± 0.11	NA	0.2
T. amyloid vol. (mL/m^2^)	57 ± 27	53 ± 22	NA	0.7
T. cell vol. (mL/m^2^)	47 ± 15	61 ± 19	NA	0.04

*: AL vs. ATTR patients

$: *p* < 0.005: Healthy subjects and CA patients

£: *p* = 0.4: Healthy subjects and CA patients

#### Mapping images

**Fig. 1:**
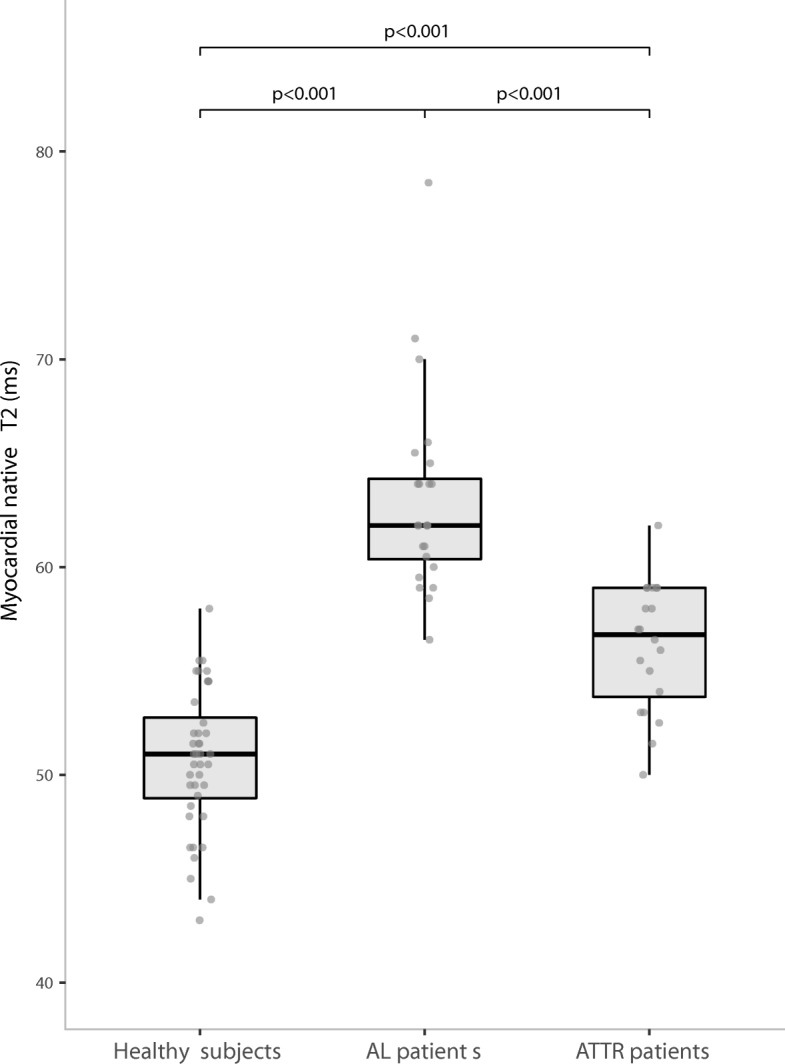
Graph shows myocardial native T2 values in healthy subjects, patients with AL amyloidosis and patients with ATTR amyloidosis. Results are shown as boxplots, with each box representing the interquartile range (1st to 3rd quartile, IQR), the line within the box indicating the median, and the whiskers extending to 1.5 times the IQR above and below the box; the dots represent individual values for each patient. Myocardial native T2 is significantly (*p* < 0.0001) higher in patients than in healthy subjects. Among patients, myocardial native T2 is significantly (*p* < 0.001) higher in AL than in ATTR patients

**Fig. 2:**
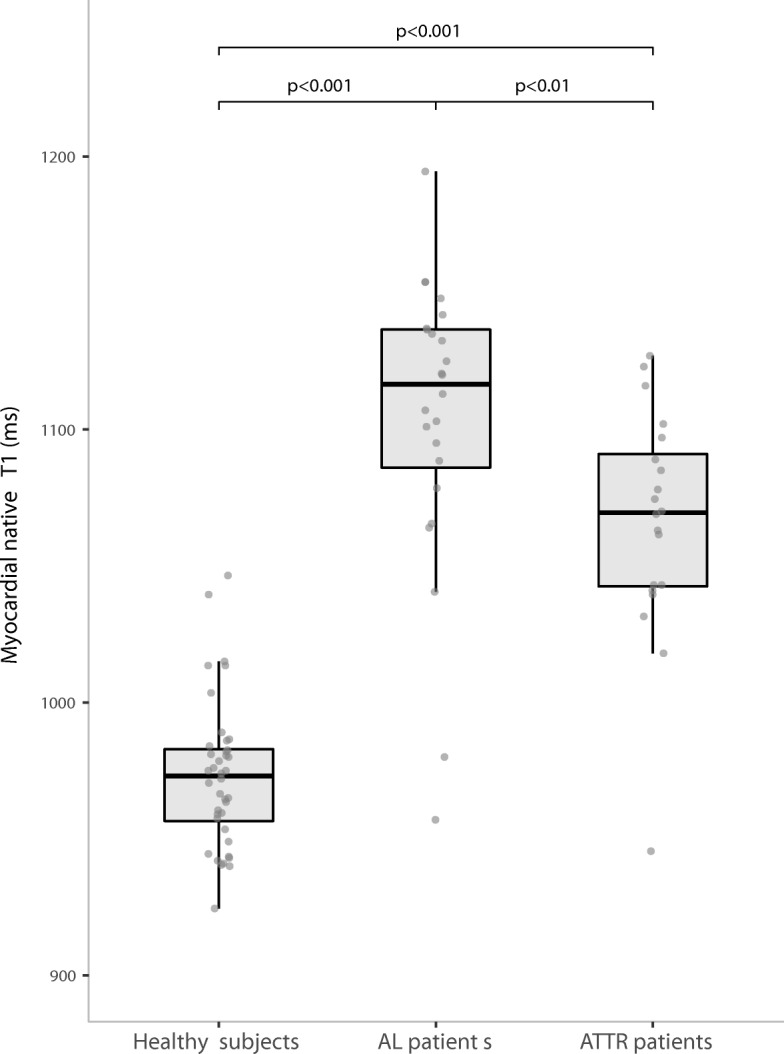
Graph shows myocardial native T1 values in healthy subjects, patients with AL amyloidosis and patients with ATTR amyloidosis. Results are shown as boxplots, with each box representing the interquartile range (1st to 3rd quartile, IQR), the line within the box indicating the median, and the whiskers extending to 1.5 times the IQR above and below the box; the dots represent individual values for each patient. Myocardial native T1 is significantly (p < 0.001) higher in patients than in healthy subjects. Among patients myocardial native T1 is significantly (*p* < 0.01) higher in AL than in ATTR patients

**Fig. 3:**
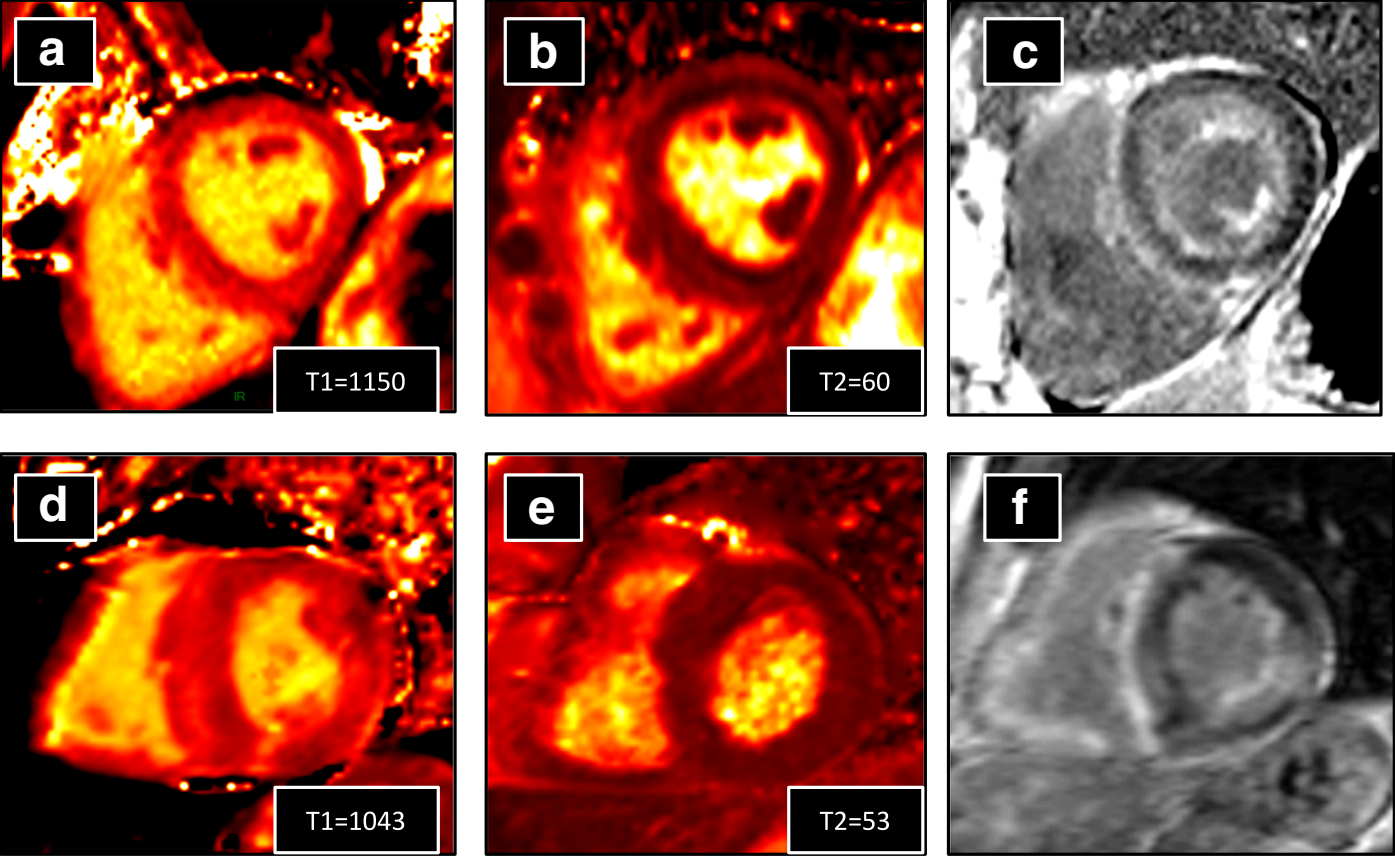
Examples of MOLLI native T1 maps (**a**, **d**), T2 maps (**b**, **e**) and LGE images (**c**, **f**) obtained in one patient with AL amyloidosis (first line) and one patient with ATTR amyloidosis (second line). AL patient exhibited higher values of myocardial native T1 and T2 (1150 and 60 ms, respectively for T1 and T2) than ATTR patient (1043 and 53 ms, respectively for T1 and T2). Both patients exhibited diffuse myocardial enhancement of left and right ventricles on LGE images

Myocardial native T2 was significantly higher in AL and ATTR patients than in healthy subjects *p* < 0.001 for both). A significant raise of myocardial native T1 was also noticed in CA patients (AL (1104 ± 54 ms); ATTR (1066 ± 42 ms)) compared to healthy subjects (975 ± 26 ms; *p* < 0.001) (Figs. 1 and 2). Examples of patients are reported in the Fig. 3. When considering the difference between AL and ATTR patients, we reported that T2 was significantly (*p* < 0.001) higher in AL (63.2 ± 4.7 ms) than in ATTR (56.2 ± 3.1 ms) patients. A raise of myocardial native T1 was also observed in AL patients (1104 ± 54 ms) in comparison to ATTR patients (1066 ± 42 ms), but in a less significant manner (*p* < 0.01). AL and ATTR patients did not exhibit significant difference regarding other mapping parameters (post contrast myocardial T1, ECV, ICV and Total amyloid volume) except for total cell volume that was significantly higher in ATTR (61 ± 19 mL/m^2^) than in AL patients (47 ± 15 mL/m^2^; *p* = 0.04). T2 ratio did not exhibit significant differences (*p* = 0.2) between groups (1.31 ± 0.4, 1.41 ± 0.2 and 1.44 ± 0.3, respectively for AL patients, ATTR patients and healthy subjects). All data are reported in Table 2.

#### Relationship between myocardial native T2 and other parameters

**Fig. 4:**
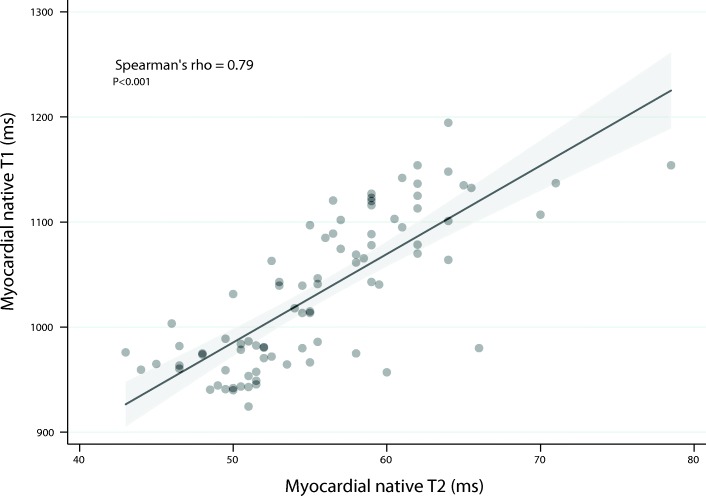
Graph shows the relationship between myocardial native T1 and myocardial native T2 in the overall population (Spearman’s rho = 0.79; *p* < 0.001). The dots represent individual values for each patient. The solid line represents the fitted regression line along with its 95% confidence interval

Myocardial native T2 was strongly correlated with myocardial native T1 (Spearman’s rho = 0.79; p < 0.001) (Fig. 4) and less markedly correlated with indexed LV mass (Spearman’s rho = 0.36; *p* = 0.001), Total cell volume (Spearman’s rho = − 0.38; *p* = 0.03) and LVEF (Spearman’s rho = − 0.26; *p* = 0.02).

### ROC-curve analysis

**Fig. 5:**
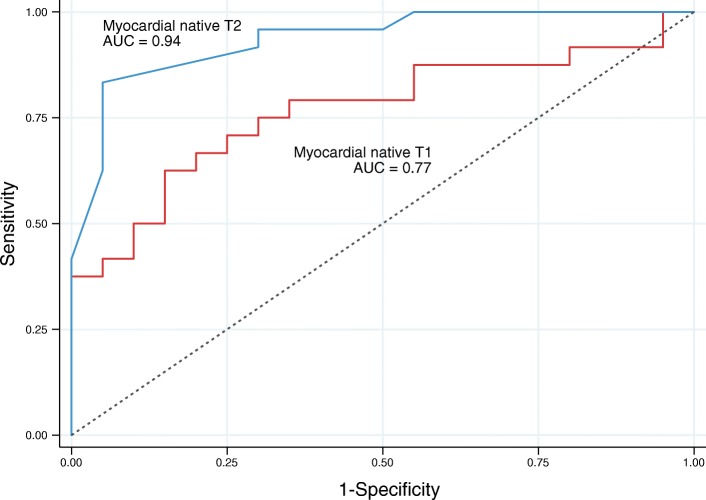
Receiver operating characteristic curves for myocardial native T2 and T1

AL patients formed the positive case group and ATTR patients were considered as negative cases. Healthy subjects were excluded to reflect clinical practice. The area under curve (AUC) of myocardial native T2 for diagnosed AL patients as opposed to ATTR patients was 0.94 (95% CI: 0.86–1, *p* < 0.001), higher than that of T1 (0.77 (95% CI: 0.62–0.91, *p* = 0.03)) (Fig. 5). For QALE score, the AUC to distinguish between ATTR and AL patients was 0.64 (95% CI: 0.48–0.81, *p* = 0.1) With a cut-off of 59.2 ms, the sensitivity and specificity of myocardial native T2 were 83 and 95%, respectively. One hundred percent of specificity was obtained with a cut-off of 63 ms (sensitivity 42%). Lower performances were obtained using T1: with a cut-off of 1092 ms, the sensitivity and specificity of myocardial native T1 to diagnose AL patients were 71 and 75%, respectively. One hundred percent of specificity was obtained with a cut-off of 1130 ms (sensitivity 37%). Myocardial native T1 and T2 exhibited similar performances to distinguish between patients and healthy subjects (AUC = 0.95, (95% CI: 0.90–1, *p* < 0.001).

### Survival analysis

**Fig. 6:**
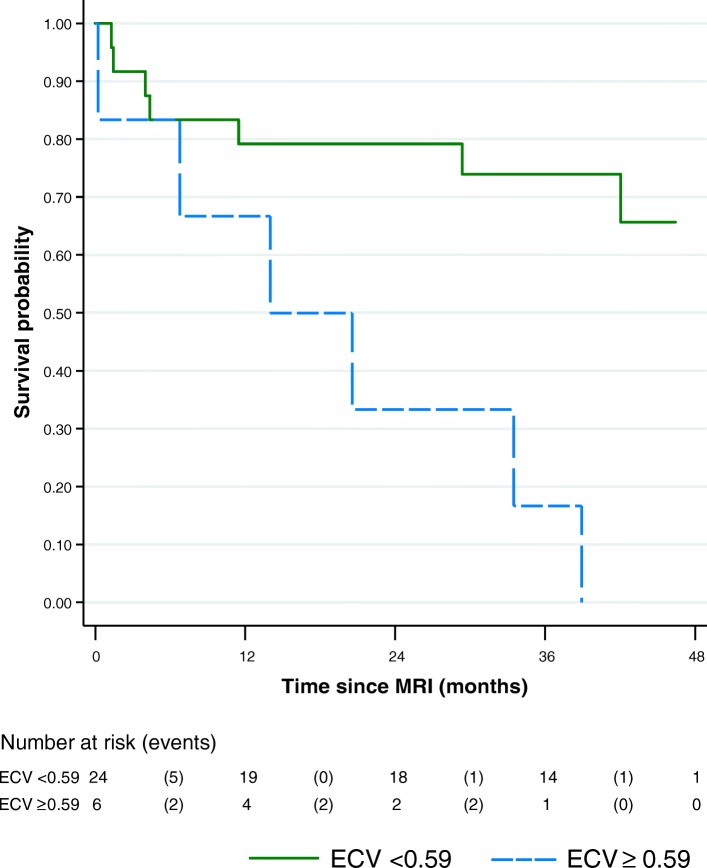
Kaplan-Meier curve for overall survival according to ECV value. A median value of 0.59 was the best predictor of survival: log-rank test *p* = 0.004; Unadjusted HR 5.33 (1.70–16.71)

Survival analysis was performed in the cohort of 42 patients with CA. In total, 18 of 42 patients died during a median follow-up of 27 months (interquartile range: 14–40 months). Myocardial native T2 was not significantly associated with overall survival in patients with CA (HR 1.03 (0.94–1.12); *p* = 0.53). Univariate Cox regression identified the following predictors: ECV (HR 1.66 per 0.1 increase in ECV (1.24–2.22); *P* = 0.0006), IVS thickness (HR 1.18 per 1 mm increase in IVS thickness (1.02–1.37); *P* = 0.02), LVEF (HR 0.64 per 10% increase in LVEF (0.47–0.87); *p* = 0.005) and post contrast myocardial T1 (HR 0.89 per 10 units increase in T1 value (0.84–0.95); *p* = 0.0005). For ECV, a value of 0.59 was identified by recursive partitioning as the optimal cutoff to predict overall survival: HR 5.33 (1.70–16.71), *p* = 0.004. Kaplan-Meier curve for patients according to ECV level is shown in Fig. 6.

## Discussion

In this study, we reported a significant raise of myocardial native T2 relaxation time suggesting myocardial edema in patients with CA, and more pronounced in AL compared to ATTR patients. Myocardial native T2 exhibited higher performances than T1 to differentiate AL and ATTR amyloidosis but was not a predictor of survival.

The question of variation of myocardial T2 in CA remains controversial in the literature with few studies addressing this topic. Using T2 mapping sequence, Sparrow et al. [18] did not find significant differences between patients with CA and controls whereas Wassmuth et al. [19] and our team [17] reported that the T2 ratio was lower in patients with CA and independently associated with shortened survival. Recently Fontana et al. showed a greater elevation of T1 (and lower ECV) in AL than in ATTR patients suggesting that myocardial edema, induced by the toxic effect of AL amyloid fibrils on cardiomyocytes, may explain this difference [22]. The significant raise of myocardial native T2 that we reported here confirms this hypothesis of myocardial edema in AL patients. Interestingly, we also detected a less intense but significant myocardial edema in ATTR patients, suggesting that the toxic effect of amyloid fibrils is probably a general phenomenon in amyloidosis. However, we did not detect a significant correlation between T2 and troponin level in favor of a direct toxicity of amyloid proteins on cardiomyocytes. From a clinical point of view, myocardial native T2 exhibited good performances to separate AL and ATTR amyloidosis in our study (AUC = 0.94), higher than T1 (AUC = 0.77) and QALE LGE score (AUC = 0.65), suggesting that it could be used as an additional marker to differentiate these 2 types of CA whose management is different. Although potentially performant to differentiate AL and ATTR, T2 does not seem to be a good marker of survival in our study, in contrast to ECV that deeply influences survival. The prognostic impact of ECV in amyloidosis was reported in several studies [16, 28, 29].

Surprisingly, we did not find in this study a significant reduction of T2 ratio in CA patients in comparison to healthy subjects, as previously reported by our group [17] and others [19]. We have no formal explanation for these results. It is possible that diffuse skeletal muscular edema exists in amyloidosis and may alter the relevance of T2 ratio by balancing more or less the increase of myocardial signal on T2 images [30]. In addition, the T2 STIR sequence is known to be sensitive to artifacts and is accompanied by a lower level of diagnostic confidence than T2 mapping as previously reported. In this work, the significance of the T2 map was higher than those observed in the previous studies on the T2 ratio supporting the impression of reliability of the T2 mapping.

In the light of our results, one can also deduce that the raise of myocardial T1 in cardiac amyloidosis is not only a simple consequence of passive amyloid proteins deposit, but is also driven by myocardial edema. The high correlation between myocardial native T1 and T2 that we noticed suggests a reciprocal influence and reinforces this idea. Therefore, because of the dual origin of its signal (interstitium and myocytes), caution should be taken to use solely T1 as a direct marker of amyloid burden and as a surrogate endpoint in drug development [31]. Its variations should be analyzed at the light of ECV value that is the purest measure of amyloid burden [8].

ATTR patients exhibited higher indexed LV mass and total cell volume in comparison to AL patients in our report, suggesting that extracellular deposition of amyloid proteins was associated with a concomitant cell hypertrophy in ATTR. These results are in line with Fontana et al. who reported a 18% increase of the intracellular space in ATTR vs. AL patients [22]. Nevertheless, we did detect significant difference of ECV and total amyloid volume between AL and ATTR in contrast to the aforementioned study that reported a significant raise of these values in ATTR patients, suggesting a more extensive amyloid deposition. We also failed to reproduce the results of Dungu et al. regarding differentiation of AL and ATTR using LGE sequences [21]. The QALE score was slightly higher in ATTR than in AL patients but did not exhibit significant difference as previously reported. Similarly, we did not observe systematic RV involvement in ATTR patients, as described by Dungu et al. [21].

### Study limitation

First, the number of subjects included in this work was relatively limited and control group was younger than AL and ATTR patients, a difference that may influence (weakly) measurements of myocardial native T1 with the MOLLI sequence [32]. Second, we did not include other causes of LV wall thickening such as hypertrophic cardiomyopathies, aortic stenosis or hypertensive cardiomyopathies. Third, calculation of myocardial T1 and T2 was not performed on the entire LV. The basal part of the LV, more commonly affected in CA, was also not measured specifically using a short-axis basal section. Finally, the measurements of myocardial T1 and T2 included both LGE + and LGE- myocardial areas; focal variations of T1 and T2 maps related to amyloid proteins deposit were not studied.

## Conclusion

In this study, we reported a significant raise of myocardial native T2 in AL patients in comparison to ATTR patients. T2 separated effectively AL and ATTR patients and could be considered as an additional marker to distinguish these 2 types of CA. Although more intense than in AL amyloidosis, myocardial edema was also detected in ATTR patients suggesting that myocardial edema plays a role in the raise of myocardial native T1 in CA. As opposed to ECV, T2 did not seem to impact survival of CA patients.

## Abbreviations

AL: Light chain
ANOVA: Analysis of variance
ATTR: Transthyretin
AUC: Areas under curve
bSSFP: Balanced steady state free precession
CA: Cardiac amyloidosis
CMR: Cardiovascular magnetic resonance
ECG: Electrocardiogram
ECV: Extracellular volume fraction
EMB: Endomyocardial biopsy
HR: Hazard ratios
ICV: Intracellular volume
IVS: Interventricular septum
LGE: Late gadolinium enhanced
LV: Left ventricle/left ventricular
LVEF: Left ventricular ejection fraction
MOLLI: Modified Look-Locker inversion recovery
PSIR: Phase sensitive inversion recovery
QALE: Query amyloid late enhancement
ROC: Receiver operating characteristic
ROI: Region-of-interest
RPA: Recursive partitioning analysis
RV: Right ventricle/right ventricular
STIR: Short tau inversion recovery
TI: Inversion time
